# Supplementary material for: BBS8-dependent ciliary Hedgehog signaling governs cell fate in the white adipose tissue
Source: EMBO J. 2025 Aug 20;44(19):5315–36. doi: 10.1038/s44318-025-00524-y (PMC12489102; doi:10.1038/s44318-025-00524-y)
Supplement: Supplementary file 1 — Appendix [file 44318_2025_524_MOESM1_ESM.pdf]

Appendix

Table of Contents

Appendix Figure S1	page 2
Appendix Figure S2	page 3
Appendix Figure S3	page 4
Appendix Figure S4	page 6
Appendix Figure S5	page 8
Appendix Figure S6	page 10
Appendix Table S1	page 12

Appendix Figure S1

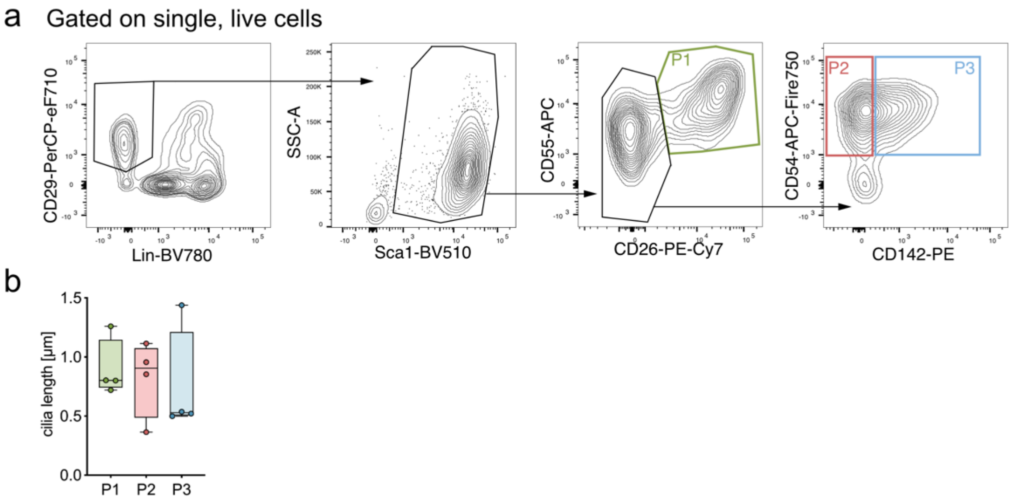

Fig. S1. Primary cilia regulate the adipogenic potential of adipocytes precursors subpopulations (APCs). a, Simplified gating strategy to identify the three APC subpopulations using flow cytometry. **b**, Ciliary length of the three subpopulations measured by the ARL13B mask via CiliaQ. Each data point represents one animal (n = 4, >4 cilia per n), box plots show the 10th to 90th percentiles (box), with whiskers extending to the minimum and maximum values and the horizontal line representing the median. Cells were isolated from wild-type mice at 7-9 weeks.

Appendix Figure S2

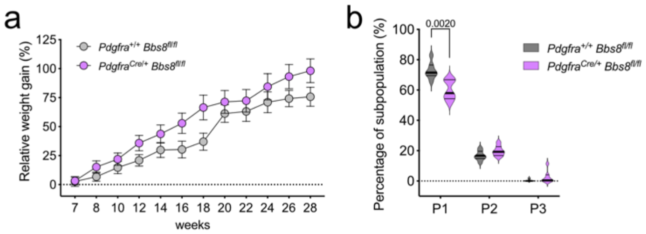

Fig. S2. Loss of BBS8 in APCs results in obesity. a, Relative body weight of chow diet-fed *Pdgfra*$^{+/+}$*Bbs8*$^{flox/flox}$ and *Pdgfra*$^{Cre/+}$ *Bbs8*$^{flox/flox}$ mice. Weights were normalized to the mean body weight at 9 weeks. Data are shown mean ± SEM, p-values were determined using a Two-way ANOVA with repeated measurements (mixed models). Post-hoc p-value correction for multiple testing was performed using Bonferroni adjustment (n = 9). **b,** Frequency distribution of P1-P3 from the total APC pool from *Pdgfra*$^{+/+}$ *Bbs8*$^{flox/flox}$ and *Pdgfra*$^{Cre/+}$ *Bbs8*$^{flox/flox}$ mice (n = 7), isolated at 6-7 weeks (lean state). P-values were determined using unpaired Student's t-test.

Appendix Figure S3

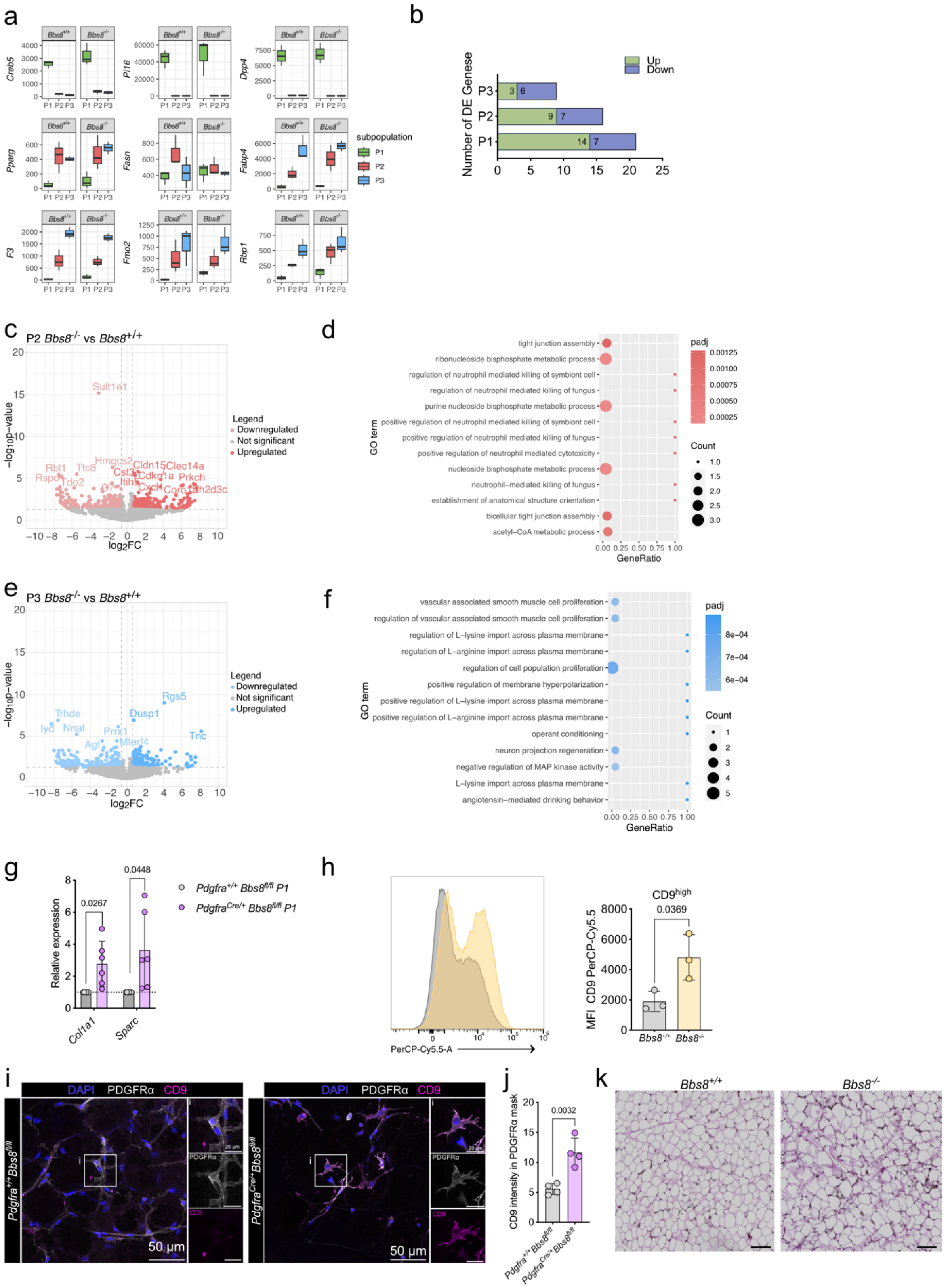

Fig. S3. Phenotypic changes in APCs of lean *Bbs8*$^{-/-}$ mice (5-7 weeks). a, Expression level of published P1, P2, and P3 markers from *Bbs8*$^{-/-}$ and *Bbs8*$^{+/+}$ mice, isolated at 7-8 weeks (lean state) (n = 3). Data are shown as mean ± SD. **b,** Differentially expressed genes (DEG) from *Bbs8*$^{-/-}$ and *Bbs8*$^{+/+}$ mice, isolated at 7-8 weeks (lean state) (n = 3). Benjamini-Hochberg

method was used to calculate multiple testing adjusted p-values. **c,** Volcano plots depicting the DEGs for P2 APCs from (**b)** (n = 3). **d,** Over-representation analysis (ORA) of upregulated DEGs, highlighting the biological processes from gene ontology analysis in P2 *Bbs8*$^{-/-}$ compared to *Bbs8*$^{+/+}$ APCs. Values were calculated using the Fisher's exact test (FDR < 0.05). **e,** Volcano plots depicting the DEGs for P3 APCs from (**b)** (n = 3). **f,** Over-representation analysis (ORA) of upregulated DEGs, highlighting the biological processes from gene ontology analysis in P3 *Bbs8*$^{-/-}$ compared to *Bbs8*$^{+/+}$ APCs. Values were calculated using the Fisher's exact test (FDR < 0.05). **g,** Median Fluorescent Intensity (MFI) of CD9-PerCP-Cy5.5 signal of PDGFRα^{+} cells from *Bbs8*$^{+/+}$ and *Bbs8*$^{-/-}$ mice (left). Histogram showing CD9-PerCP-Cy5.5 signal from concatenated *Bbs8*$^{+/+}$and *Bbs8*$^{-/-}$ files (right). Data are shown as mean ± SD, p-values have been determined using an unpaired Student's t-test (n = 3). **g**, Expression level of selected fibrosis marker genes in *Pdgfra*$^{+/+}$*Bbs8*$^{flox/flox}$ and *Pdgfra*$^{cre/+}$*Bbs8*$^{flox/flox}$ P1 APCs, isolated at 5-6 weeks (lean state). Each data point represents one animal (n = 5). Data are shown as mean ± SD, p-values have been determined using a one-sample t-test. **i**, Whole mount staining of gWAT of *Pdgfra*$^{+/+}$*Bbs8*$^{flox/flox}$ and *Pdgfra*$^{cre/+}$*Bbs8*$^{flox/flox}$ mice at 7 weeks (lean state). Stained with DAPI (blue), PDGFRα (white) to label APCs, and CD9 (magenta). Scale bar = 50 µm, 20 µm. **j**, Quantification of CD9 mean intensity in a PDGFRα mask depicted in (**i**). Each data point represents a technical replicate from n = 1 mouse, p-value was determined using an unpaired Student's t-test. **k**, Sirius Red and Elastin-van-Giesson staining of gWAT from lean *Bbs8*$^{+/+}$ and *Bbs8*$^{-/-}$ mice (7 weeks). Scale bar = 50 µm.

Appendix Figure S4

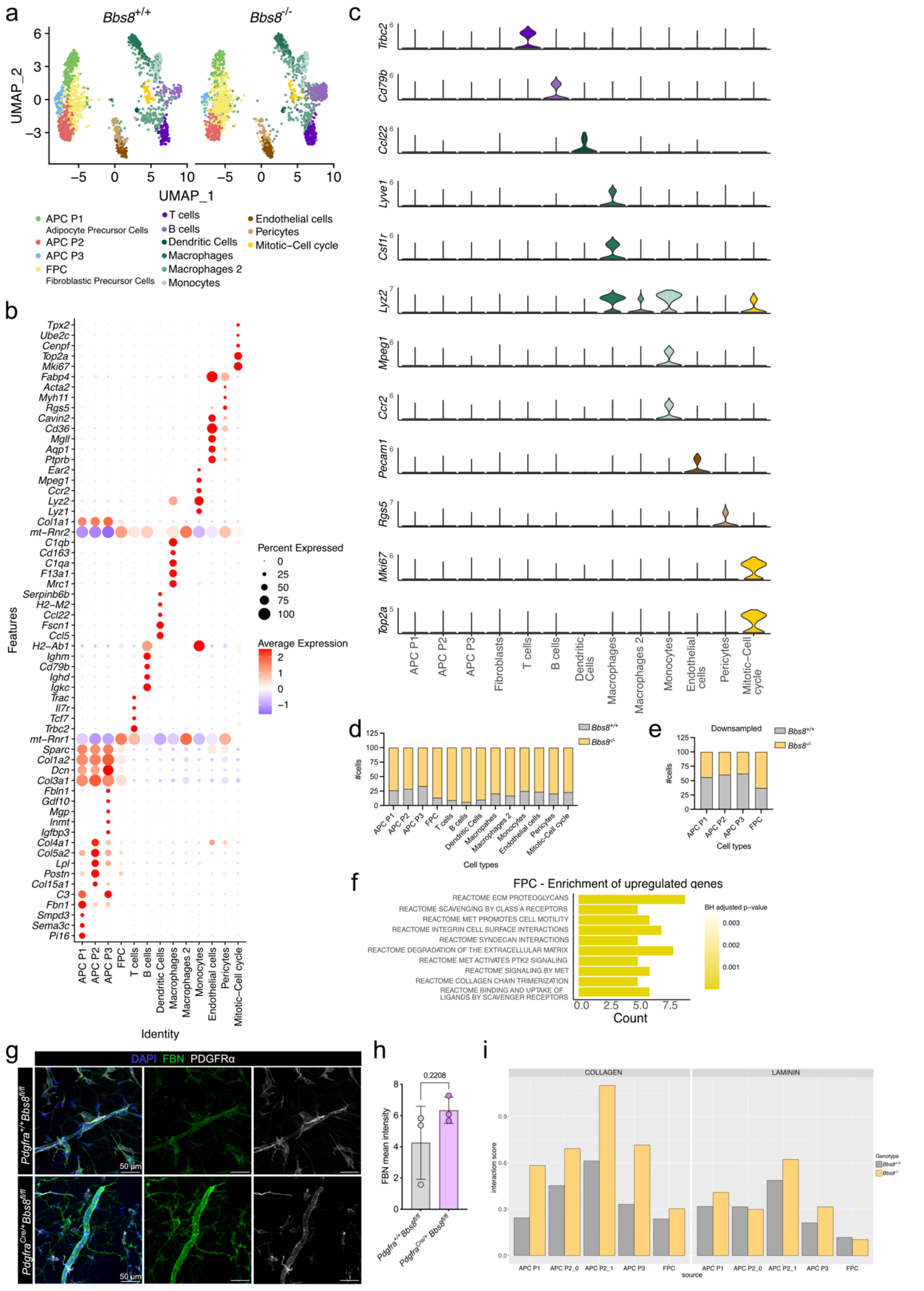

Fig. S4. Loss of *BBS8* drives P1 cells into fibrogenic cells. a, UMAP plot analysis on iWAT SVF from *Bbs8*$^{+/+}$ and *Bbs8*$^{-/-}$ mice, isolated at 7-8 weeks (lean state), split per genotype. **b,**

Dot plot for top 5 marker genes in all cell clusters determined in Fig. S4a. **c,** Violin plots of published markers for all cell clusters determined in Fig. S4a**. d,** Relative cell numbers in each cluster per genotype. **e**, Relative cell numbers for the subsetted APC P1-P3 and FPC cluster. *Bbs8*$^{-/-}$ cells were down sampled. **f**, Reactome pathway analysis from genes upregulated in the FPC cluster. **g,** Whole mount staining of gWAT of *Pdgfra*$^{+/+}$*Bbs8*$^{flox/flox}$ and *Pdgfra*$^{cre/+}$*Bbs8*$^{flox/flox}$ mice at 7 weeks (lean state). Stained with DAPI (blue), anti-Pdgfrα antibody (white) to label APCs, and anti-Fibronectin antibody (FBN, green). Scale bar = 50 µm. **h**, Quantification of the mean intensity of FBN from (**g)**. Each data point represents a technical replicate from n = 1 mouse, p-value was determined using an unpaired Student's t-test. **i,** Interaction of APCs and endothelial cells via collagen and Laminin pathways is increased in *Bbs8*$^{-/-}$ APCs. Collagen and Laminin interaction scores in the APC subclusters interacting with endothelial cells.

Appendix Figure S5

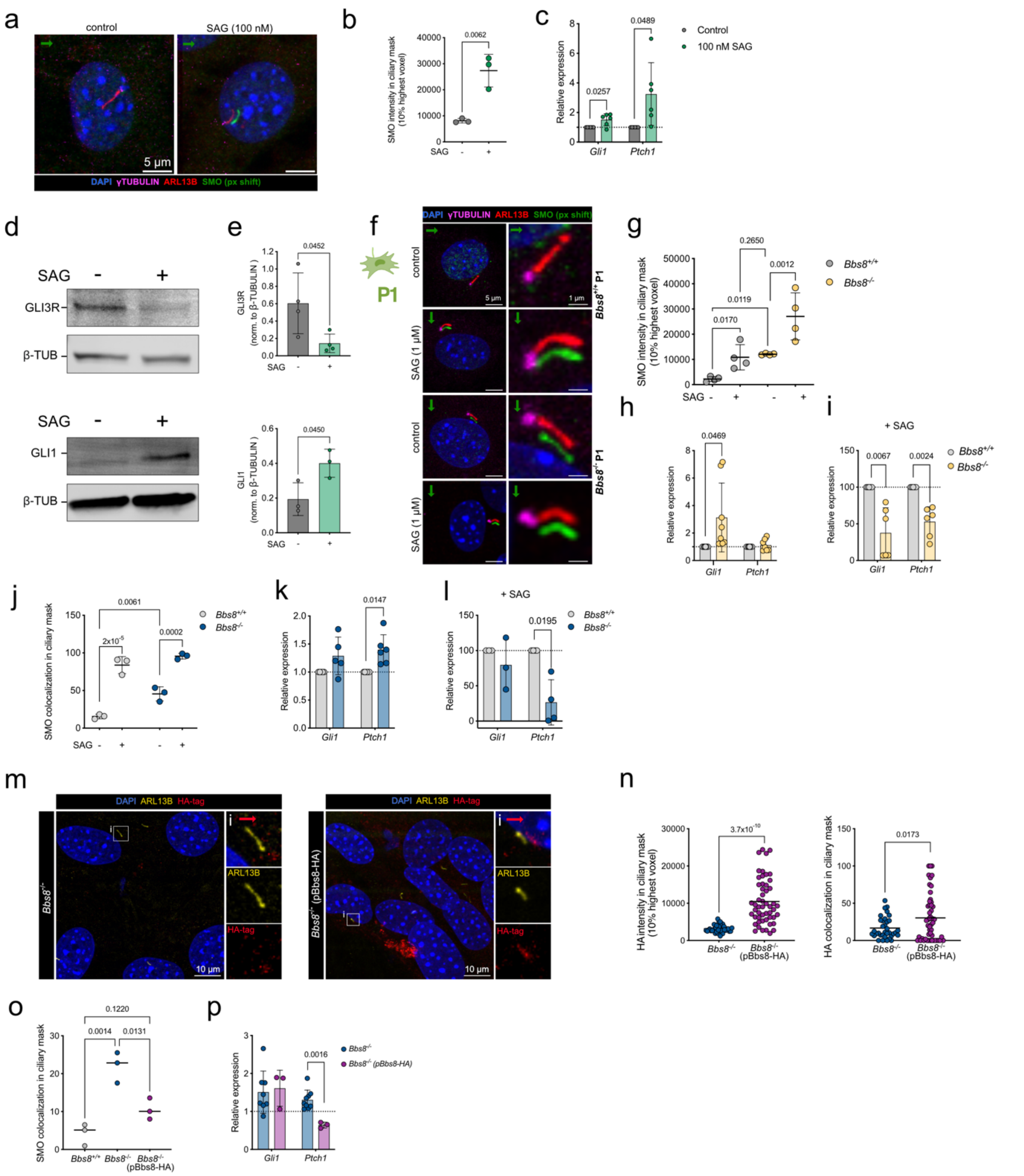

Fig. S5. Loss of BBS8 results in ectopic Hedgehog (Hh) signaling and diminishes the Hh-dependent signaling response. a, Fluorescence confocal images of 3T3-L1 labeled against Smoothened (green, SMO, 5 px shift indicated by the green arrow), ARL13B (red, cilia), γ-Tubulin (magenta, basal body), and with DAPI (blue). Cells were treated with H_2O (control) or 100 nM SAG for 24 h. Scale bars are indicated. **b**, Quantification of the ciliary SMO localization shown in (a). Each data point represents a biological replicate (n = 3; >20 cilia per n). **c**, Relative mRNA expression of *Gli1* and *Ptch1* in 3T3-L1 cells after treatment with 100 nM SAG for 24 h assessed by qRT-PCR. The expression was normalized to the vehicle control. **d**, Western Blot analysis of GLI transcription factors GLI1 and GLI3R of 3T3-L1 cells after treatment with 100 nM SAG for 24 h. β-Tubulin was used as a loading control. **e,** Quantification of GLI3R and GLI1 by normalizing the band intensity to the loading control (β-Tubulin). Each

data point represents a biological replicate (n = 3-4); p-values have been determined using an unpaired Student's t-test. **f**, Fluorescence confocal images of APC P1 cells isolated by magnetic separation from *Bbs8*$^{+/+}$ and *Bbs8*$^{-/-}$ mice, isolated at 7 weeks (lean state), labeled against Smoothened (green, SMO; 5 px shift indicated by the green arrow), and ARL13B (red, cilia), g-Tubulin (magenta, basal body), and with DAPI (blue). Cells were treated with H_2O (control) or 1 µM SAG for 24 h. Scale bars are indicated. **g,** Quantification of the ciliary SMO localization in APC P1 cells. Each data point represents one animal (n = 4; >4 cilia per n), p-values have been determined using one-way ANOVA. **h-i,** Relative mRNA expression of *Gli1* and *Ptch1* in APC P1 from *Bbs8*$^{+/+}$ and *Bbs8*$^{-/-}$ mice at (**h**) basal level or (**i**) after treatment with SAG (1 µM) for 24 h assessed by qRT-PCR. The expression was normalized to *Bbs8*$^{+/+}$ expression values. Each data point represents >10 cilia from one animal (n = 6-8). All data are shown as mean ± SD, p-values have been determined using a one-sample t-test. **j**, Quantification of the ciliary SMO localization in mouse embryonic fibroblasts (MEF). The colocalization of SMO pixels in the ciliary (ARL13B) mask is depicted. Each data point represents >10 cilia from one animal (n = 3). Each data point represents one animal. All data are shown as mean ± SD, p-values have been determined using a Two-way ANOVA. **k-l,** Relative mRNA expression of *Gli1* and *Ptch1* in MEFs from *Bbs8*$^{+/+}$ and *Bbs8*$^{-/-}$ mice at (**k**) basal level or (**l**) after treatment with SAG (1 µM) for 24 h assessed by qRT-PCR. The expression was normalized to *Bbs8*$^{+/+}$ expression values. Each data point represents one animal (n = 5-6). All data are shown as mean ± SD, p-values have been determined using an unpaired Student's t-test. **m**, Overexpression of HA-tagged BBS8 in *Bbs8*$^{-/-}$ MEFs. Transfection was assessed by fluorescent antibody staining for the HA-tag (red, 5 px shift in insets is indicated by the arow). **n,** Quantification of (m). Each data point represents a cilium (n = 3; >6 cilia per n); p-value was determined by an unpaired Students t-test. **o,** Quantification of the ciliary SMO localization in non-transfected *Bbs8*$^{+/+}$ and *Bbs8*$^{-/-}$ MEFs, and transfected *Bbs8*$^{-/-}$ MEFs. Each data point represents a biological replicate (n = 3; > 5 cilia per n); p-values were determined by one-way ANOVA. **p,** Relative mRNA expression of *Gli1* and *Ptch1* in non-transfected *Bbs8*$^{-/-}$ MEFs and transfected *Bbs8*$^{-/-}$ MEFs assessed by qRT-PCR. The expression was normalized to *Bbs8*$^{+/+}$ expression values (shown as the dotted line). Data are shown as individual values (dots) and mean (bars) ± S.D. Different n are indicated by dots; p-values were calculated by an unpaired Student's t-test. Each data point represents one biological replicate (n = 3-9), p-values have been determined using an unpaired Student's t-test.

Appendix Figure S6

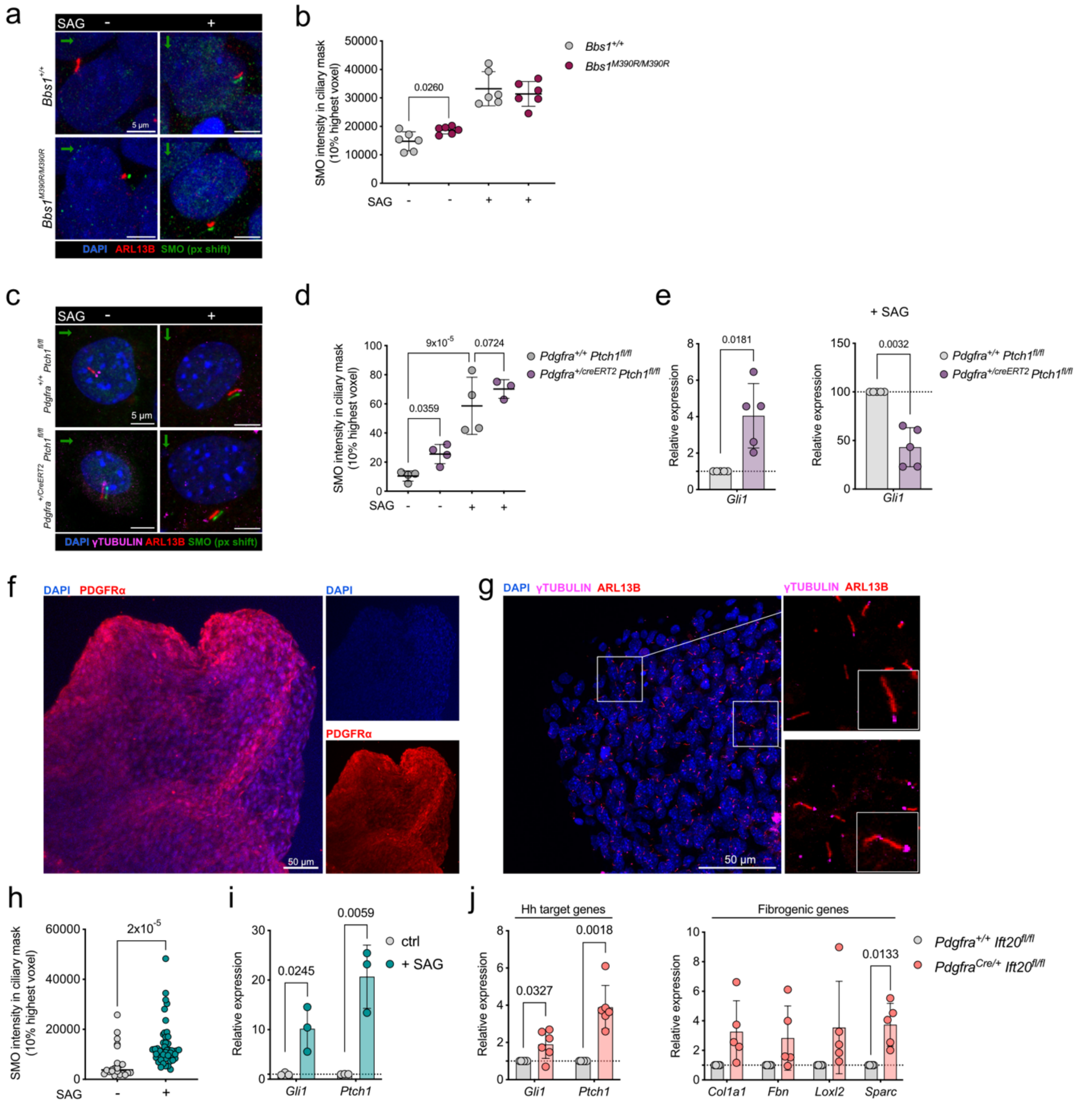

Fig. S6. Hedgehog (Hh) phenotype in different ciliopathy models. a, Fluorescence confocal images of *BBS1*$^{+/+}$ and *BBS1*$^{M390R/M390R}$ iPSCs, labeled against ARL13B (red, cilia) and Smoothened (green, SMO, 5 px shift indicated by the green arrow), and with DAPI (blue). Cells were treated with H_2O (control) or 1 µM SAG for 24 h. **b,** Quantification of the ciliary SMO localization in *BBS1*$^{+/+}$ and *BBS1*$^{M390R/M390R}$ iPSCs. Each data point represents a technical replicate from n = 2 biological replicates (> 100 cilia per image); p-value has been determined using an unpaired student's t-test. **c,** Fluorescence confocal images of APCs isolated from *Pdgfra*$^{+/+}$ *Ptch1*$^{fl/fl}$ and *Pdgfra*$^{+/CreERT2}$ *Ptch1*$^{fl/fl}$ mice at 5-7 weeks of age and treated with 10 nM tamoxifen for 48 h. Cells were stained for ARL13B (red, cilia), γ-Tubulin (magenta, basal body), and SMO (green, 5 px shift indicated by the green arrow), and with DAPI (blue). **d,** Quantification of ciliary SMO localization shown in **(c)**. Each data point represents one animal (n = 4; >35 cilia per n), p-values have been determined using an unpaired Student's t-test. **e,** Relative mRNA expression of *Gli1* in *Pdgfra*$^{+/+}$ *Ptch1*$^{fl/fl}$ and *Pdgfra*$^{+/CreERT2}$ *Ptch1*$^{fl/fl}$ cells after 48 h of 10 nM tamoxifen treatment, assessed by qRT-PCR. The expression values were normalized to *Pdgfra*$^{+/+}$ *Ptch1*$^{fl/fl}$ levels. Each data point represents one animal (n = 5), p-values have been determined using a one-sample t-test. **f,** Gonadal WAT was harvested from P4 male mice, and whole mount staining against PDGFRα was performed to label APCs. **g**, At P4, APCs are all ciliated visualized by labeling against ARL13B (red, cilia) and γ-Tubulin (magenta, basal body). **h,** Quantification of the ciliary SMO localization in P4 gWAT APCs after treatment with 1 µM SAG or vehicle control (H_2O) for 24 h. Each data point represents

one cilium from one tissue (> 20 cilia per image), p-values have been determined using an unpaired students t-test. **i,** Relative mRNA expression of *Gli1* and *Ptch1* in P4 gWAT APCs after treatment with 1 µM SAG or vehicle control (H_2O) for 24 h assessed by qRT-PCR. The expression was normalized to control treated expression values. Each data point represents one tissue (n = 3), p-values have been determined using an unpaired students t-test. **j,** Relative mRNA expression of Hh target genes (left) and fibrogenic genes (right) in P4 precursor tissue from *Pdgfra*$^{+/+}$ *Ift20*$^{fl/fl}$ and *Pdgfra*$^{Cre/+}$ *Ift20*$^{fl/fl}$ mice. Expression was normalized to expression values of *Pdgfra*$^{+/+}$ *Ift20*$^{fl/fl}$. Each data point represents a biological replicate (n = 5-6), p-values were calculated by a one-sample t-test. Scale bars are indicated. All data are shown as mean ± SD.

Appendix Table S1, Primer sequences

Gene name	Fwd sequence	Rev sequence
Col1a1	GAGATGATGGGGAAGCTGGC	CTCGGTGTCCCTTCATTCCG
Col5a1	CTTGTCCGATGGCAAGTGGC	CATCATCCAGAATCCGGGAGC
Col6a1	CAGGTACTACCGGTGTGACC	GAAGTACTTGACCGCATCCAC
Loxl2	CTGCCTGGAGGACACTGAGT	CGGTGATGTCTATCCACTGGC
Fibronectin	CTCCGAGACCAGTGCATCG	GAATCTTGGCACTGGTCAATGG
Sparc	CTGTGCCGAGAGTTCCCAG	CAGCAACTTCAGTCTGCTGAG
Gli1	TACCATGAGCCCTTCTTTAGGA	GCATCATTGAACCCCGAGTAG
Ptch1	GCCTTGGCTGTGGGATTAAAG	CTTCTCCTATCTTCTGACGGGT
Tatabp	GAGCTCTGGAATTGTACCGCAG	CATGATGACTGCAGCAAATCGC
Gapdh	AGGTCGGTGTGAACGGATTTG	TGTAGACCATGTAGTTGAGGTCA
